# Older Migrants and Overcoming Employment Barriers: Does Community Activism Provide the Answer?

**DOI:** 10.3389/fsoc.2022.845623

**Published:** 2022-04-28

**Authors:** Matt Flynn, Louise Wong

**Affiliations:** ^1^Centre for Research Into the Older Workforce, University of Hull, Hull, United Kingdom; ^2^Wai Yin Society, Manchester, United Kingdom

**Keywords:** older worker, migration, ageism, intersectionality, community activism

## Abstract

As populations age and pension ages rise, there is a growing interest in the ability of workers to extend working life. In response to a call for a more robust dialogue on the heterogeneity of the older workforce, this article explores the interplay between different employment barriers faced by one group facing significant employment barriers: older migrants. Older Chinese migrants in the United Kingdom face multiple barriers to work resulting from age, ethnicity and the intersectionality of such barriers which creates a unique set of barriers to continued work. Community activism can play an important role in supporting older constituents, particularly in matching the skills which they have to offer with the needs within and beyond the migrant community. In this study, we use Participatory Action Research to explore with older Chinese migrants the barriers they face in the job market and how community activism can empower them in maintaining employment. As government seeks to raise real retirement ages, more research is needed on its implications for vulnerable groups of older people including migrants.

## Introduction

In response to a growing debate on work, age and retirement, Taylor et al. (2016) called for a critical research agenda on aging workplaces which they argued is needed so that social policy can better respond to demographic change. They noted that much of the literature has treated the older workforce as a homogenous labor group ‘obscuring the qualitatively different needs, motivations and desires of older workers' (p. 681). For social policy to respond to the welfare and employment needs of older workers, a more heterogenous and socially embedded approach to research is needed, with a particular focus on those at risk of poverty and social exclusion. They noted that qualitative research can help to better understand how the relationship between work and age is shaped by factors like work experiences and life circumstances (p. 685). In this article, we respond to their call by focusing on the experience of older migrants whose labor market place is shaped by age, ethnicity and life-course.

From a social policy perspective, there are valid reasons for focusing attention on older migrants. First, the growing level of both internal and cross-national migration requires a policy framework for addressing risks of poverty and exclusion faced by a range of migrant populations (Torres, 2019). Migrants are at risk of economic disadvantage and social immobility (Gorodzeisky and Semyonov, 2011) and have less access to both state and occupational pension entitlements than non-migrants (Han, 2013) and those who need to work longer face significant barriers when it comes to the interplay of ageism and migration status (Dwyer et al., 2018). Further, migrants in the West have different experiences of work and retirement than the native population. For example, they are at risk of health-related early retirement (Escribá-Esteve et al., 2012) and often plan retirement around spending time in both their native and host countries (Ciobanu and Hunter, 2017). At the same time, they are more likely to be in work beyond State Pension Age, primarily owing to a lack of pension or other savings toward retirement. In the UK, for example, migrants 65–70 years old are more likely than UK-born to be in work (30 vs. 22%) and less likely to consider themselves retired (51 vs. 68%).^1^ Almost one in five 65–70-year-old migrants are either seeking work or wanting to work but economically inactive due to a health problem or caring responsibility. Migrants, including older ones, also play an essential role in the infrastructure of social care and support for aging populations (Repetti et al., 2021). Government led programmes to promote active aging- including the promotion of sustainable work in later life- may be inhibited by older migrants' precarious work context, lack of access to employer support and inaccessibility of social benefits (Ciobanu et al., 2017).

Community activism has played an important role in terms of social and economic provision for migrants as well as addressing gaps in public policies and welfare (Letiecq and Schmalzbauer, 2012). Community groups can empower members through collective action, representation and shared welfare (Mainwaring et al., 2020). The aim of this article therefore is to look at the role of community organizing in overcoming barriers faced by older migrants in attaining sustainable work. Working with a community group of mostly economic migrants from Hong Kong and China in the UK city of Manchester, known as the Wai Yin Society (henceforth Wai Yin), we have used Participatory Action Research (PAR) along with expert interviews within the organization to explore with older migrants themselves their work and retirement. We seek to answer three questions:

- How do older Chinese immigrants' lifetime experiences in and out of work create a unique set of barriers to sustainable work?
- What support do older Chinese migrants need to prepare for and maintain sustainable retirements and to what extent does continued work play a role?
- Does community activism offer a way to provide such support?

## Literature Review

The focus of our study are people who migrated early in life and have “aged in place” (Warnes et al., 2004, p. 311), a group which is described as,

“A lifetime of disadvantage and deprivation, including poor health care and housing conditions, few opportunities to learn the local language, and very often the insults of cultural and racial discrimination” (ibid: p. 312).

“Aged in place” migrants therefore can be characterized as not only facing present-day economic and social disadvantage, but also the legacy of lifetime disadvantage which, within the context of work, limits access to sustainable employment, training to support skills development and a “social safety net” in terms of state and employer benefits in order to maintain economic status during periods of illness and hardship. Further, the construct focuses on place-making (i.e., shared experience of disadvantage or deprivation) rather than ethnicity, race or culture (which may be shared by some but not all aged in place migrants (Johansson et al., 2013). Consequently, the term is particularly relevant to studies like this one which explore how labor market exclusion impacts on later life employment.

### Older Migrant Workers and Disadvantage

Older migrants' career trajectories are often marked by precariousness, social disadvantage, economic illness and hardship (Chin, 2019). Further, older migrants face discrimination not only in terms of age, but also potentially based on ethnicity, disability and gender (Stypińska and Gordo, 2018). Here, it is important to draw lessons from the literature on intersectionality to recognize that the barriers which older migrants face may be unique and separate from those of both older non-migrant workers and younger migrants. It is also an important and useful tool for understanding how economic and social experiences create distinct experiences of Aged in Place migrants which are separate from other migrant groups.

McBride et al. (2014) noted that the intersectionality methodological approach can be an important tool for understanding how multiple discrimination unfold in terms of marginalization beyond their cumulative and respective effects. This is particularly the case with regards to older migrants. Different generations of economic migrants have experiences with discrimination and marginalization which are unique from one another. Further, different forms of exclusion based on migration status, gender and disability, for example, can have compounding effects impacting both everyday experiences and the impact of policy solutions formulated to address issues of disadvantage (Molloy et al., 2003).

Older migrants' career trajectories may also diverge from those of non-migrant workers because of their concentration in self-employment (Clark et al., 2017). There have been two reasons suggested for the high level of self-employment. First, self-employment is a remedy to employment barriers resulting from language problems or difficulties with regards to skills transferability (Abada et al., 2014). Second, self-employment is a product of “neighborhood enclaves” in which community members are connected through shared culture and language as well as access to finance (Klaesson and Öner, 2020). Migrants may choose to live within neighborhood enclaves in order maintain ethnic identity as well as avoid factors leading to social exclusion (Chilingaryan, 2011). However, it has also been suggested that residing in ethnically concentrated urban areas may have a negative impact on migrants' employment prospects, as it reduces labor seeking mobility (Clark and Drinkwater, 2002) and that lower employment prospects and earning potential are the compensating differential for explaining the cultural value migrants seek from living in ethnically concentrated areas (Talen, 2018).

### Community Activism

Next, we turn to community activism as a way to overcome barriers which older migrants face to employment and three ways in which community groups are important:

First, community activism can play a practical role of communications within the population, for example by helping migrants to claim social benefits. A report for the HM Revenue and Customs (Radu et al., 2010) recommended that the agency engage with community organizations to address the low levels of social welfare claims amongst migrants. The report noted that such engagement could help not only in terms of disseminating information, but also in addressing social norms which prevent migrants from claiming entitlements. More recently, community groups have played a crucial role in disseminating COVID transmission mitigation measures (Fan, 2021) and ensuring workplace risk assessments have been carried out in sectors with high levels of migrant employment (Moore et al., 2021).

Second, community activism can be a vehicle for political representation. Such political representation is realized through both engaging with external institutions (e.g., government and NGO's) and mobilizing political action within the community. This advocacy is important in overcoming political inequalities which migrants face as well as gaining self-representation against the native population (Ku, 2010). Torres (2019) observed a blind spot in terms of political representation as a vehicle for social justice for migrants as they age, noting that political and economic social justice are complementary.

Finally, community activism plays an important role in promoting social cohesion between community members in order to address shared issues and mobilize political and community action (Matthews and Astbury, 2017). It has been noted that community activism is particularly important in addressing problems of social exclusion which are resulting from multiple forms discrimination through the organization and mobilization of the community across social and demographic boundaries (Pero, 2014).

It is for these three interlocking reasons that we are focusing this paper on the role of community activism in addressing the barriers which older migrants face in maintaining employability. Community organization can both help migrants negotiate changes in public policy and social welfare aimed at encouraging delayed retirement as well as acting as a voice for a constituency whose labor market position makes extended working lives particularly difficult and contentious.

## Methods

The project was carried out using PAR to conduct research with rather than on participants (Bradbury Huang, 2010). In particular, there are four main benefits which this approach brings to a research project of this kind. First, the approach facilitates thick description of participants' tacit knowledge and understandings (Reason and Bradbury, 2001). The methodology focuses the researcher(s) on a rigorous enquiry into participants' practical and experiential knowledge through a process. Accordingly, we are able to achieve the objectives of thick description as set by Geertz (1973) of prolonged engagement, triangulation, member checking and holistic processes. Second, the methodology requires a juxtaposition of the researcher's and participants' frames of reference in a process that results in changed perspectives of both stakeholders (Cho and Trent, 2006). Third, the epistemological approach has aspirations for not only producing technical and explanatory value to participants, but emancipatory outcomes as well (e.g., new pathways to sustainable work for older migrants). This is consistent with an ontological approach which recognizes the human actor as a knowledgeable agent, aiming for catalytic validity (Reason and Rowan, 1981) in which the validity of the research is assessed according to its capacity to change understanding of all participants and is therefore a change agent. Finally, PAR acknowledges the ethical dimension of research, and the ownership individuals have over their own thoughts and knowledge (Eikeland, 2006). In keeping with this approach, we refer to our participants as co-researchers. As Heron and Reason described,

“Everyone is involved in the design and management of the inquiry; everyone gets the experience and action that is being explored; everyone is involved in making sense and drawing conclusions; thus everyone involved can take initiative and exert influence on the process” (Heron and Reason, 2006, p. 144).

In this case, in 2015, we worked with eight members of the Wai Yin community who responded to our invitation for 50+ people to participate in a project to explore issues of employability of older Chinese migrants like themselves. We revisited the findings with Wai Yin executives in 2021 and discuss advancements on the community group's work on the subject in the results section. The primary aim of the project was practical- we were aiming to learn from Wai Yin members themselves what the community organization could do to support older members. However, we were also exploring wider issues of the meaning which they attach to work and career as well as both their expectations (for those in work) and experiences in retirement and how they may differ from those of British people. Finally, we aimed to explore with the group how they view government policy with regards to pensions and retirement and how it impacts on older migrants.

Group discussions occurred five times over a 3-month period. Each meeting occurred for a 2-h period. The discussions were taped and material such as flip charts were retained. Informed consent from participants was attained. In addition, field notes were written up and circulated to the group at least 1 week ahead of each meeting. At the beginning of each meeting, the field notes were discussed and amended based on the group consensus. All of the data- both audio and written material- was input and analyzed using NVIVO software. We conducted thematic analysis with the research questions framing the top-level coding and sub-themes emerging through on-going reflexive dialogue (Braun and Clarke, 2006) between the two co-authors (an academic and practitioner). Group participants and interviewees were given the opportunity to read and comment on this article prior to submission.

In order to gain a better understanding of how community activism could support older migrants like our co-researchers, four Wai Yin executives, organizers and managers were interviewed to gain understanding about the background of the organization; the challenges facing the community, particularly older and retired members. These interviews were meant to gain an understanding of how the community group both interfaced with actors such as government, businesses and other community groups; and organized and mobilized constituents in order to address challenges facing the Chinese community. Tables 1, 2 provides the list of group participants and interviewees using pseudonyms.

**Table 1:** Co-researchers in our work and retirement group.

**Name**	**Age**	**Gender**	**Status**	**Contributions**
Abe	55	Male	Self-employed	Businessman owning a Chinese nursing home
Betsy	58	Female	Employed	In work caring for elderly
Carl	63	Male	Employed	Second generation Chinese family member. Works for NHS
Doug	73	Male	Retired	Retired from catering. Married to Eloise
Eloise	70	Female	Retired	Retired from catering. Married to Doug
Frances	62	Female	Volunteer	Retired from catering. Volunteer
Georgia	63	Female	Volunteer	Retired from administration work. Volunteer
Henry	72	Male	Volunteer	Retired from catering. Volunteers as a gardener

**Table 2:** Wai Yin executives, managers and organizers.

**Name**	**Role**
John	Health and social care manager
Margaret	Chief executive
Jane	Finance manager
Judith	Organizer

The final output of the research was a report to Wai Yin, jointly presented to the executive board. This journal article is a second output which has been jointly written by one academic and one Wai Yin co-researcher.

## Results

In this section, we will discuss the course of the group research on the employability of older migrants. What follows is a narrative shaped by three sources: the co-researchers themselves, executive interviews (jointly carried out by the group as a whole), and literature assembled by us on older workers and age management. The triangulation of sources is consistent with the role of the PAR facilitator in surfacing discrepancies between conventional wisdom (e.g., as articulated by stakeholder groups like employers, unions and professional associations) and the understandings of their own contexts (Wadsworth, 2006). As we will discuss below, the co-researchers identified three spheres in which their experiences in work diverge from those of non-migrant older workers: (1) the impact of life-course on present labor market placement; (2) multiple barriers to sustainable work; and 3) the support needed to find and maintain sustainable work.

### Work, Retirement and Being Blown Off Course With Late-Career Plans

We started our workshops by discussing the expectations and experiences of older Wai Yin members regarding work. The ideal planned life trajectory of a Wai Yin member was described as running a family business until the last child finished university:

“Work hard and save to send your children to university. Once the last child has finished uni, sell the restaurant and use the money to live off in retirement” (Carl).

Respondents had discussed how major social and economic disruptions like recessions or more recently the COVID pandemic can blow older migrants' retirement plans off course. For self-employed migrants, macro-economic conditions can impact their ability to sell their business and generate enough wealth to retire. Additionally, male participants described feeling displaced within the community since they lacked the financial resources with which to invest in younger people's businesses. It was described how men would often use the wealth generated by their businesses to invest in those of younger community members, while women would devote time to volunteering both with Wai Yin and other groups. A financial shortfall therefore broke the cycle of reciprocity within the “neighborhood enclave.” Abe noted that older men want to stay in work so as not to “lose face” within the community.

Wai Yin has developed programmes to support constituents into work and to address employability problems faced by Chinese migrants such as the transferability of skills which had been attained abroad.

“The problem was that they [Wai Yin community members] could do the work but not the qualifications and so employers didn't want to know. We tried to bridge that gap” (Margaret).

Career development programmes which Wai Yin had developed for younger migrants were unsuitable for older people who lacked both current formal skills and employment histories working for employers. Further, needing to delay retirement was seen by many older Wai Yin members as “losing face,” as late retirement was described as a signal that the older person had inadequately prepared for retirement. As Frances noted,

“If you are older than 65, it is more difficult to find a job elsewhere. It is easier to run your own business because you are not going to tell yourself off by being too old but if you go to work for somebody else they would probably think that you are too old” (Frances).

For our co-researchers as well as other Wai Yin members, major economic and social disruptions can lead to older migrants' retirement plans, to paraphrase (Vickerstaff et al., 2004, p. 26), “blown off their retirement course.” Property prices fell, and as a result, couples were unable to generate enough wealth to retire comfortably. Their options were described as limited. One of the participants noted that some business owners continue to run their restaurants with the hope of selling up once the economy eventually improves. However, another made the point that many older restaurant owners are worn out before they reach the age of sixty and continuing in the work they do is too physically demanding. Co-researchers said that older people avoid asking their children to help run the business, as for many their aims are to help their children build lives and careers outside of the catering industry, education was described as he only mean of claiming up the social ladder and build their lives away from take away shops or working in restaurants. At one point in the group discussion, for example, one of us asked Henry whether, when he experienced an injury which had limited his mobility, he had asked his children to help with kitchen work which he had been unable to carry out. It was explained:

“Our dream is to get our children to university and out of the restaurant business because we know how hard it is and we want our children to have better lives than ours […] We don't want to ask them back” (Henry).

Selling the business and working for an employer was also seen as difficult. It was noted that, for those who had started their businesses young, the lack of an employment history, past employers from whom references could be obtained, formal qualifications and career based training made finding a job in one's fifties very problematic. Further, the lack of English language proficiency created barriers to even low paid work.

“I do think the people over 50 if they are Chinese if they have language barriers it is really difficult for them no matter what job. It is difficult for them because the language skill is not very good and they have to engage in the community. They are not confident to go out to work with people speaking the language they don't really know” (Jane).

Older Wai Yin community members whose retirement plans were affected by the 2008 financial crisis were therefore left with the prospect of retiring with a smaller nest egg than they had anticipated. Further, they lacked routes back into work.

### Intersectionality of Multiple Barriers to Work

Next, we discuss the multiple forms of discrimination identified by our co-researchers. We start with the two forms of stereotypes (age and ethnicity) which were identified by Rhee et al. (2013). It was noted that employers tend to favor young people, who are perceived as cheaper and more flexible sources of labor and non-migrant workers who have stronger English proficiency. Older migrants were therefore described by our co-researchers as the last choice for employers.

“It's not so bad for the younger generation. A company will take on a 19-year-old trainee who they can pay £6.19 an hour. Why would they want a 50-year-old, especially one who has trouble reading and writing English?” (Betsy).

However, it was not simply double barriers which they faced, but rather the intersection of being both older and having been born overseas. For example, while both younger and older migrants commonly face language proficiency difficulties, the former have more opportunities to improve their English skills through training and language exchange programs. Further, the current generation of Chinese young people moving to the UK are better educated than those who immigrated decades before. For those older people who had not had the opportunities to improve their English skills early in life, language difficulties and social isolation became mutually perpetuating. As one older person described when discussing work in a restaurant,

“If you can't communicate well, you stay in the kitchen away from the customers. That's why the husband can't speak as well as the wife. The husband will stay in the back preparing the food, while the wife will greet customers and talk with everyone. So, when we get to our age, it's easier for me to go into town and talk to others than him [her husband]” (Eloise).

Another co-researcher added that employers tend to be less supportive of older job applicants with poor English proficiency than younger ones because it is assumed that a person who has lived in the UK for a long time will have had the opportunity to master the language. She discussed the experience of a friend who had applied for a job as an office cleaner. During the interview, the friend was asked to read a toilet cleaner bottle label.

“She couldn't pronounce most of the words. Nobody can. Why is it necessary for the job?” (Betsy).

Two other dimensions of intersectionality played out in our discussions. The first concerned how the onset of health problems and associated disabilities played out for older migrants. Work in the catering industry is often physically demanding, particularly for men who are mainly responsible for the manual work. However, because many migrants are either working in self-employment or precarious work, they cannot rely on employers to provide support should a health condition occur. Georgia, for example, discussed the experience of a contemporary who was pushed out of catering work by stress and applied for an administrative job through a recruitment agency.

“So, I went with her interview for real, what is her plan for looking for a job. She was expecting they will give her some training or an application. Then they look at the screen and says, you have any disability or long-term illness and she says, yes I have a mental health problem. It was terrible. It shocked me and I felt hurt as she felt. So I thought, no, this is not the way” (Georgia).

Second, the group discussed how issues of employability and retirement played out differently for men and women in the Wai Yin community. Both co-researchers and Wai Yin organizers cited an increase in the community of the divorce rate in the last decade, especially amongst those in their late fifties. Many older women are facing difficulties obtaining divorce settlements which would provide adequate retirement income, particularly where the family wealth was tied into a business. While pension poverty is a well-documented problem in the UK (Ebbinghaus, 2021), a Wai Yin organizer reported it as a particularly significant issue which case managers are managing because many of their older female clients lack the requisite national insurance contributions to claim the state pension and, because they have mainly worked in self-employment or precarious work, they also lack occupational pensions.

Older migrants identified four main barriers to continued employment faced by people like them within the Chinese community. Two had been identified by Rhee et al. (2013): language and discrimination; and two were new: their lack of employment history owing to years spent in either self-employment or precarious work; as well as the lack of planning for extended working life due to disruption of their life trajectories as a result of the financial crisis. Given the context, we spoke with Wai Yin managers and organizers about the services which the organization is providing older constituents who need support in finding work.

### Community Activism

Wai Yin offers help in CV writing, career advice, locating training in local colleges and apprenticeships. However, the clients of these services are almost exclusively young people. Older people rarely attend drop in sessions to ask for help because their perception of work is often physically demanding manual work which they are too worn out to do.

“They tell me they don't want to work anymore […] ‘I've been washing dishes since I came to this country. Why do you ask me to wash dishes again” (Judith).

The organization's first intervention in these situations is to help the client claim social welfare benefits to which they are entitled. However, it was noted first that clients often want or feel they need to retire well before the State Pension Age; and second may have interrupted histories of paying into National Insurance which then affects their entitlements. Further, several Wai Yin managers noted that clients who retire early are at risk of social isolation.

“When you are 50, you can go to another job when you retire because you have the language skill. But Chinese men and women can't. So, they become lonely men and women” (Judith).

The community organization had tried unsuccessfully to encourage local employers to employ older Chinese people. Wai Yin organizers did, however, recognize a need for older volunteers in the community. In particular, the organization needs people to provide interpreting services as well as outreach care for vulnerable community members. Two examples were highlighted. First, Chinese people with mental health issues are reluctant to ask for support from health care professionals because of the cultural stigma attached. The organization established a drop-in center deploying volunteers, many of them older and/or recently retired to work with health care professionals in advising and supporting clients. The benefit of deploying volunteers, according to the organizers, was that it challenged the negative perceptions of people with mental health issues which were pervasive in the community. The second project which was mentioned involved the delivery of care for Chinese people with cancer. The project, set up in partnership with MacMillan Cancer Foundation, and aimed to help families navigate through the UK health and social service systems in order to access support in caring for their relatives. Clients had language difficulties and older volunteers acted as interpreters.

According to Wai Yin organizers, their aims when recruiting older volunteers is 2-fold. First, they assess the volunteer's skills and talents and how they can be deployed to the benefit of the community. Organizers identified volunteers who were contributing to the Wai Yin community by for example training others in Tai Chi or leading gardening groups. Some of the volunteers with whom we spoke discussed how their volunteering led them to discover “hidden talents” which they enjoyed sharing with others.

Second, they aim to find ways in which volunteers can benefit from their time volunteering. This is primarily focused on addressing issues of social isolation, particularly just after retirement. Organizers discussed a number of projects such as luncheon clubs and away days which were developed to reduce loneliness in the community. According to organizers, Chinese society attaches significant cultural importance toward caring for the elderly which is usually met within the extended families. People in their fifties and sixties normally expect to spend some time caring for elderly relatives, as well as grandchildren. If the extended family is not local, people whose children have left home feel at a loss, and come to Wai Yin to take on a caring role which would normally be provided by the family,

“The idea that you volunteer your time…is different among Chinese…We don't have a problem with people coming to help the elderly because there is a strong identification that you respect your elders” (John).

Wai Yin has developed two programmes to support older migrants (both Chinese and members of other BAME groups) who are displaced from work mid-career. First, the community group was commissioned by the Manchester City Council to help deliver domiciliary care services to elderly Chinese people. Like many councils, Manchester's is facing significant constraints in the delivery of social care services, and delivery of services to elderly people with limited English skills has been particularly problematic.

Organizers in Wai Yin had sought to recruit the group's members who had recently retired to deliver care services to elderly constituents. The problem that the group faced was not that they lacked potential volunteers- in fact there was a surplus of people wanting to participate. Rather, volunteers did not have the necessary qualifications to provide eldercare. In the UK, social care workers need to achieve formal qualification through the National Vocational Qualification (NVQ) programme which involves training and assessment of on-the-job skills as matched to the occupational standards of the candidate's profession. The NVQ program was developed in large part to give workers who had derived their skills through workplace experience pathways to formal and transferable qualifications.

“What we do is that you know the NVQ training now this is the Level II and then you have the assessor. So the assessor is assessing them. So we wanted to train up, as long as we've got this link, so we can set up the NVQ trainer, this is the assessor, to Chinese assessor to assess them” (Jane).

According to Wai Yin organizers, there is a great amount of interest amongst older people in the community to gain NVQ's. The problem stems from the program's emphasis on language skills. In order to complete the assessment, candidates need to not only show assessors how they carry out their work, and thereby demonstrate competency, but also verbally narrate the work which they are completing. Non-native speakers struggle when communicating in English, especially when describing complex tasks.

“The English requirement is a big and unnecessary block on Chinese people who want an NVQ […] They can do the job. They just can't talk through what they're doing” (John).

To address this barrier, organizers in Wai Yin have adapted the NVQ program to tailor it toward the needs of its constituents. First, it developed training programs which takes modules from the national curriculum and modified them in part to be delivered in Chinese, but also to focus modules more on demonstrating competency rather than communicating processes. They worked with a local college as well as charity groups representing people with learning disabilities.

“We work with those charities that deal with learning disabilities because the people they deal with have, invariably, communication difficulties. They can't write so staff have learnt ways of communicating in visual terms” (John).

Second, Wai Yin organizers have trained Cantonese speaking examiners to assess candidates. They also developed strategies which non-native speaking candidates to prepare for assessments by first writing out their work processes in Cantonese and then translating their written work into English so that they can communicate with more confidence.

According to organizers, the NVQ program has the potential to achieve three objectives for the local community. First, it can help mobilize talent within the community to deliver sought after eldercare services which are not now being provided by the public sector.

“They [trained care workers] would work for Wai Yin as well as others as well. So if they got the qualification, if they have confidence enough in speaking English a lot of support worker will require NVQ qualification anyway” (Jane).

This is an important goal for the community group, as the number of elderly migrants is rapidly growing as the first generation to arrive in the UK ages. Second, organizers are developing methods for delivering vocational based training to people who have historically been excluded from the formal educational system. The group's aspiration is not only to deliver NVQ training in social care, but also to support migrants in gaining formal qualifications based on training they may have received from abroad. Third, organizers noted that candidates who have gone through the program have developed strategies for communicating in English which have then helped them in job interviews.

Second, in partnership with the Manchester based radio station AllFM, Wai Yin has developed a programme to provide radio broadcasting skills for volunteer participants in a programme called Dragons Voice (an award winning programme recognized by the Queen's Award for Voluntary Service, National Community Radio Awards, and High Sheriff Special Recognition Award). Dragons Voice provides a medium of expression for Manchester's Chinese community to talk about their experiences living in the UK. Dragons Voice Radio explores issues such as isolation and loneliness, identified problems in migrant communities (Ambition for Ageing, 2020). Broadcasting in Cantonese and English, it encourages those with limited English skills to contribute. The development of Dragons Voice was supported by the Greater Manchester Mental Health NHS Foundation Trust, initially to encourage those in the Manchester Chinese community to access mental health services and promote positive mental health. The shows and its presenters and have grown and evolved since its inception to explore a range of issues affecting their community, promote Chinese Culture and promote events within their community. Working with ALLFM, Dragons Voice provides training to volunteers who participate in the radio shows. Training covers all aspects of radio production including writing, editing, literacy, team working and software skills. These are transferrable skills that can be utilized to address employability issues in older migrants. Also providing a medium for community activism, Dragons Voice gives older Chinese migrants a platform to discuss challenges of mental health, joblessness and isolation living in the UK (Ambition for Ageing, 2020).

Both the social care and Dragons Voice initiatives show how Wai Yin is addressing joblessness in its older community in three ways. First, the organization is tailoring employability support to the particular set of problems older migrants face in seeking reemployment: namely language barriers, interrupted career trajectories and lack of formal and transferable skills. Second, both programmes are also addressing broader community challenges like care service shortages and public outreach over COVID. Finally, they are vehicles for political and community voice for older migrants.

## Discussion

The aims of this paper were 2-fold: First to add to the still nascent body of literature on the employment barriers faced by older migrants; and second to show how community activism can play an important role in helping older migrants find sustainable work as retirement ages rise. We selected older migrants from China and Wai Yin due to its historical establishment in organizing and providing services to the local Chinese community.

Our first research question asked what employment barriers older Chinese migrants who would be described as Aged in Place face. Earlier, we suggested that the conceptual framework of intersectionality can be instructive in understanding the labor market placement of older migrants which differentiate them from older workers generally. Our co-researchers identified three ways in which migration status and age intersect. First, precarious careers and self-employment make them less attractive job candidates with employers seeking workers with formal qualifications and trackable career histories. Second, the lack of language skills can block access to sustainable work even in jobs with which English skills are not a priority. Third, older migrants have less access to institutions like employers, public services, and trade unions which are meant to deliver support (e.g., lifelong learning, phased retirement, health initiatives) to older workers in extending working life. Accordingly, social policies in the UK and many other parts of the world concerning extending working life are failing to reach older migrants.

Our second two questions asked what support older Chinese migrants face *and* whether community organizations could contribute to delivering such support. In this case study, community activism has played three important roles for migrant groups: communication, representation and cohesion. Our first observation is that the approach which Wai Yin as a community group was different for younger than older job seekers. For the younger generation, the group offers training and drop-in centers for CV writing and interview preparation. It has also engaged with local businesses to encourage them to employ and/or provide apprenticeships for younger Chinese people who are seeking work, arguing the “business case” for doing so. In other words, the community group put communication and representation at the forefront, but rather less emphasis on cohesion. This could perhaps be explained by the observation from the group participants that older Chinese migrants often want their children to move away from the local community, at least regarding work because work in the catering industry is both physically demanding and precarious (Song, 2015).

The organization's approach to older job seekers, by contrast, started with communication and cohesion. Communication took the form of helping older constituents claim their pension entitlement, while helping them plan for retirement. Cohesion was intergenerational, but between people in their fifties and sixties who had lost some of the intrinsic benefits from work (having a purpose and social interaction) and were seeking financial security in old age with elderly Chinese migrants who needed care from providers who understood their language and culture. Through the gateway of volunteering, the organization introduced training, qualifications and employability skills which would then be a pathway into secure employment. In that way, the organization has replicated what might be considered a “neighborhood enclave” by creating work opportunities for older job seekers by identifying needs within the community.

### Limitations

We note two limitations to our study. First, the study was framed as a qualitative study and we have outlined our objectives in the methods section. However, a quantitative based study such as secondary analysis of national or international datasets like the English Longitudinal Study on Aging would provide insight into the experiences of older migrants in work and retirement and transitions between the two. As we outlined in the methodology section, PAR enables marginalized groups to work with researchers to explore and find solutions to problems they face. However, as Dedding et al. (2021) noted, even when a study is carried out over a period of time, findings are nevertheless time-bounded which in turn limits the granularity with which co-researchers can explore an issue. Further, generalizability is not possible by the small numbers of migrants and segmentation of this part of the population. Second, we have made clear that our study focuses on specific group of older migrants- aged in place Chinese migrants in the UK- and we want to emphasize that experiences and expectations of older migrants is diverse. We comment on the need for further studies in the conclusion.

## Conclusion

In terms of public policy, older migrants are part of the fabric of diversity of the older workforce (for a discussion on the heterogeneity of the older workforce, see Flynn, 2010 for a more in depth discussion) which must then be reflected in governmental “carrots and sticks” which encourage extended working lives. Older people who are in precarious work, are low skilled, have interrupted careers and/or have been subject to multiple forms of discrimination are more at risk of poverty in old age, and many “aging in place” migrants would certainly fit this description. Community activism, which has played an important role in integrating newly arrived migrants can also play an important role as they age to make their life more productive and valued their contribution during their working life.

Next, we would like to point to the value of participative action research as a methodology for research on age and work, particularly in relation to the employability of vulnerable older workers. The value of action research as a methodology with which older workers themselves can reflect upon, and therefore influence, their own careers has been mentioned elsewhere (e.g., Billett and Woerkom, 2008), but few studies have emerged in relation to work (for an exception see (Hilsen and Ennals, 2005). Much of the research on age and work tend to problematize either or both the extension of working life and/or maintenance of existing retirement ages. We began our research with the agenda of exploring the employment barriers faced by older migrants, but it was only through exploring with Wai Yin members that we were able to appreciate the meaning which they attach to meaningful work and as a consequence Wai Yin as a stakeholder is able to map out the deployment of skills with its constituency to address challenges within the Chinese community. For our co-researchers, participation in the project provided the opportunity to explore the meaning they attach to work and retirement, but also how they shape the experience of the two both on an individual and, via Wai Yin, collective basis. As Judith said,

“At the beginning, I was very confused with the idea of work after retirement because I thought they were opposites. Now I can see that the [voluntary] work I have been doing is work and I have never really retired” (Judith).

PAR can therefore make an important contribution in identifying ways of reconciling the extension of working life with active aging through what Vincent et al. (2001) has called for as research *with* rather than research *on* older people.

Finally, we note the lack of research on the experiences of older migrants, especially those who are aging in place, in terms of multiple and intersecting barriers they face not only in terms of employment but also health services and state benefits as well as avoiding social exclusion. Our research has shown that community activism can serve older Chinese migrants both in terms of providing employability support and representation within and outside the community. However, Chinese diaspora are relatively well-organized and suited to activism (Wang et al., 2021), however other ethnic communities are more disperse and with less of a collective voice on public policy matters. Further research on older migrants, especially marginalized groups, for example undocumented groups, is needed to better understand how support can be delivered and tailored to their needs.

## Data Availability Statement

The datasets presented in this study can be found in online repositories. The names of the repository/repositories and accession number(s) can be found at: https://beta.ukdataservice.ac.uk/datacatalogue/studies/study?id=851630.

## Ethics Statement

The studies involving human participants were reviewed and approved by Newcastle University Business School. The patients/participants provided their written informed consent to participate in this study.

## Author Contributions

Both authors listed have made a substantial, direct, and intellectual contribution to the work and approved it for publication.

## Funding

Bilateral (Hong Kong): Age Diversity: Applying the Capabilities Approach to Career Development Across the Life Course ES/I028072/1.

## Conflict of Interest

The authors declare that the research was conducted in the absence of any commercial or financial relationships that could be construed as a potential conflict of interest.

## Publisher's Note

All claims expressed in this article are solely those of the authors and do not necessarily represent those of their affiliated organizations, or those of the publisher, the editors and the reviewers. Any product that may be evaluated in this article, or claim that may be made by its manufacturer, is not guaranteed or endorsed by the publisher.

## Footnotes

^1^UK Labour Force Survey Q3 2021.
